# The Effect of Bariatric Surgery on Circulating Levels of Lipoprotein (a): A Meta-analysis

**DOI:** 10.1155/2022/8435133

**Published:** 2022-08-17

**Authors:** Tannaz Jamialahmadi, Željko Reiner, Mona Alidadi, Matthew Kroh, Wael Almahmeed, Massimiliano Ruscica, Cesare Sirtori, Manfredi Rizzo, Raul D. Santos, Amirhossein Sahebkar

**Affiliations:** ^1^Applied Biomedical Research Center, Mashhad University of Medical Sciences, Mashhad, Iran; ^2^Surgical Oncology Research Center, Mashhad University of Medical Sciences, Mashhad, Iran; ^3^University Hospital Centre Zagreb, Department of Internal Medicine, Zagreb, Croatia; ^4^Digestive Disease and Surgery Institute, Cleveland Clinic, Cleveland, Ohio, USA; ^5^Heart and Vascular Institute, Cleveland Clinic Abu Dhabi, Abu Dhabi, UAE; ^6^Department of Pharmacological and Biomolecular Sciences, Università degli Studi di Milano, Milan, Italy; ^7^Department of Health Promotion, Mother and Child Care, Internal Medicine and Medical Specialties (Promise), School of Medicine, University of Palermo, Italy; ^8^Lipid Clinic Heart Institute (Incor), University of São Paulo, Medical School Hospital, São Paulo, Brazil; ^9^Biotechnology Research Center, Pharmaceutical Technology Institute, Mashhad University of Medical Sciences, Mashhad, Iran; ^10^Department of Biotechnology, School of Pharmacy, Mashhad University of Medical Sciences, Mashhad, Iran

## Abstract

**Background:**

Obesity, especially severe obesity, is associated with a higher risk of atherosclerotic cardiovascular disease (ASCVD) morbidity and mortality. Bariatric surgery is a durable and effective weight loss therapy for patients with severe obesity and weight-related comorbidities. Elevated plasma levels of lipoprotein (a) (Lp(a)) are causally associated with ASCVD. The aim of this meta-analysis was to analyze whether bariatric surgery is associated with Lp(a) concentrations.

**Methods:**

A literature search in PubMed, Scopus, Embase, and Web of Science was performed from inception to May 1st, 2021. A random-effects model and the generic inverse variance weighting method were used to compensate for the heterogeneity of studies in terms of study design, treatment duration, and the characteristics of the studied populations. A random-effects metaregression model was used to explore the association with an estimated effect size. Evaluation of funnel plot, Begg's rank correlation, and Egger's weighted regression tests were used to assess the presence of publication bias in the meta-analysis.

**Results:**

Meta-analysis of 13 studies including 1551 patients showed a significant decrease of circulating Lp(a) after bariatric surgery (SMD: -0.438, 95% CI: -0.702, -0.174, *p* < 0.001, *I*^2^: 94.05%). The results of the metaregression did not indicate any significant association between the changes in Lp(a) and duration of follow-up after surgery, reduction in body mass index, or baseline Lp(a) concentration. The reduction in circulating Lp(a) was robust in the leave-one-out sensitivity analysis.

**Conclusion:**

Bariatric surgery significantly decreases circulating Lp(a) concentrations. This decrease may have a positive effect on ASCVD in obese patients.

## 1. Introduction

It is well known that obesity, especially severe obesity, is associated with a higher risk of atherosclerotic cardiovascular disease (ASCVD) morbidity and mortality [1]. Obesity is a widespread disease on a global scale and a major public health issue [2]. The use of bariatric surgical procedures has increased steadily over the past decades because these procedures result in significant and long-term weight loss, more than the one achieved by diet and lifestyle modifications alone. It is important to stress that bariatric surgery prolongs the lifespan in high-risk individuals for ASCVD [3, 4]. However, less than 2% of eligible patients have bariatric surgery despite the fact that bariatric therapies today are well established and have good safety and efficacy.

The most widely performed bariatric procedures are sleeve gastrectomy (SG), Roux-en-Y gastric bypass (RYGB), laparoscopic adjustable gastric band (LAGB), biliopancreatic diversion/duodenal switch (BPD/DS), and one anastomosis gastric bypass/minigastric bypass (OAGB/MGB) [5].

Weight loss, no matter how achieved, decreases the risk of ASCVD, cardiovascular events, and total mortality. Weight loss has beneficial effects on the main risk factors for ASCVD including elevated total and LDL-cholesterol (LDL-C), triglycerides, and decreased HDL-cholesterol (HDL-C) [6]. Bariatric surgery has beneficial effects on cardiovascular indices [7–9]. For instance, it has been shown that gastric bypass surgery improved all lipid profile parameters, although sleeve gastrectomy only improved HDL-C and triglyceride levels [10, 11]. It has also been recently demonstrated that sleeve gastrectomy causes an increase in HDL-C and that biliopancreatic diversion causes a significant decrease in total cholesterol, LDL-C, non-HDL-C, and LDL-C/non-HDL-C [12]. It has also been shown that bariatric surgery might prevent or slow atherogenesis in the early stages by breaking the vicious circle between inflammation and endothelial dysfunction [13]. Bariatric surgery also results in a decrease in pulse wave velocity (PWV), which might be used as an independent surrogate marker of ASCVD improvement [14].

Lipoprotein (a) (Lp(a)) is a cholesterol-rich LDL moiety that is covalently linked to a glycoprotein-apolipoprotein (a) ((apo (a)) by a disulfide bond [15]. Elevated plasma Lp(a) is widely accepted as a causal risk factor for myocardial infarction, atherothrombotic stroke, and calcified aortic stenosis [16–22]. Genetic findings strongly suggest that elevated plasma Lp(a), similarly to elevated LDL-C, is causally related to premature ASCVD and cardiovascular events, as well as mortality [23–26]. The accumulation of the LDL component in atherosclerotic plaque is regarded to be an important component of the atherogenic processes. Also, prothrombotic effects due to homology of apo (a) and plasminogen, as well as induction of a multilevel proinflammatory response mediated by oxidized phospholipid (OxPL), are supposed to be additional atherogenic mechanisms [27, 28]. The prothrombotic and proinflammatory effects of Lp(a) have been proposed to promote plaque instability, resulting in plaque rupture and atherothrombotic events [29]. Overall, Lp(a) plasma levels are stable, although variants in the *LPA* gene determine 30-40% of the variance [30]. However, *LPA* gene expression may be increased by inflammation, while diseases like hypothyroidism and chronic kidney disease may affect Lp(a) removal.

Considering the profound metabolic changes that occur after bariatric surgery and its effect on proatherogenic lipoproteins [12], the aim of this meta-analysis was to evaluate whether bariatric surgery could change Lp(a) concentrations. So far, no meta-analysis has been performed to analyze this issue.

## 2. Methods

### 2.1. Search Strategy

This meta-analysis was performed based on the 2009 preferred reporting items for systematic reviews and meta-analysis (PRISMA) guidelines [31]. From inception to May 1st, 2021, PubMed, Scopus, Embase, and Web of Science were searched using the following keywords in titles and abstracts (also in combination with MESH terms): (“bariatric surgery” OR gastroplast∗ OR “gastric bypass” OR “Roux-en-Y” OR “gastric band” OR “biliopancreatic diversion” OR gastrectom∗ OR “duodenal switch” OR “gastrointestinal diversion” OR gastroenterostom∗ OR “jejunoileal bypass” OR “obesity surgery” OR “weight loss surgery” OR “weight-loss surgery” OR “bariatric procedure” OR “sleeve surgery” OR “metabolic surgery”) AND (“lipoprotein (a)” OR “lipoprotein (a)” OR “Lp(a)”).

### 2.2. Study Selection

Only original peer-reviewed studies written in English were considered. All forms of bariatric surgery procedures (with or without supplemental medical therapies) which reported circulating Lp(a) levels before and after surgery were studied. The exclusion criteria were abstracts only, letters, case reports, comments, meta-analyses, duplicate studies, animal studies, reviews, non-English language papers, studies with no surgical intervention, and studies without outcomes.

### 2.3. Data Extraction

After deleting duplicate studies, two independent authors examined the titles and abstracts of the remaining papers for eligibility. The full texts of the eligible studies were collected. If two (or more) papers on the same research topic were published by the same organization and/or authors, the more recent study with a larger sample size was included. Any disagreements were resolved by authors' discussion and consensus. The following information was extracted: (1) first author's name, (2) year of publication, (3) type of surgery, (4) study design, (5) characteristics of the patients, (6) levels of Lp(a), and (7) duration of follow-up.

### 2.4. Quality Assessment

The Newcastle-Ottawa Scale (NOS) was applied to evaluate study quality in this meta-analysis [32]. Three features of each study were taken into account for this scale: (1) the selection of studied patients (4 items), (2) the comparability of studied populations (1 item), and (3) the ascertainment of exposure (3 items) in case-control studies or outcome of interest in cohort studies.

### 2.5. Quantitative Data Synthesis

This meta-analysis was performed using Comprehensive Meta-Analysis (CMA) V2 software (Biostat, NJ) [33]. Information regarding sample size, means, and standard deviations from each group was extracted to calculate the standardized mean differences (SMDs). SMDs were applied because several different types of assays were used to determine plasma Lp(a) levels. Random effects meta-analysis was used to estimate the effect size. The heterogeneity of studies regarding treatment duration, study design, and the characteristics of the studied populations was determined using a random-effects model (owing to interstudy heterogeneity) and the generic inverse variance weighting approach [31]. When the outcome measures were reported as median and range (or 95% confidence interval (CI)), mean and SD values were computed by the approach described by Hozo et al. [34]. When only standard error of the mean (SEM) was reported, SD was computed using the following formula: SD = SEM × sqrt (*n*), where “*n*” represents the number of participants. To analyze the influence of each study on the overall effect size, a sensitivity analysis was done using the leave-one-out approach (i.e., deleting one study each time and repeating the analysis) [35, 36]. Statistical heterogeneity between the trials was evaluated using Cochran's *Q* test and *I*^2^ statistic as a measure of variability.

### 2.6. Metaregression

A metaregression analysis was performed to investigate the impact of changes in body mass index (BMI) and postsurgery follow-up duration with the estimated effect size of surgery on Lp(a) concentrations.

### 2.7. Publication Bias

To investigate the presence of publication bias, the funnel plot, Begg's rank correlation, and Egger's weighted regression tests were used. When funnel plot asymmetry was detected, potentially missing studies were inserted using the “trim and fill” approach. In case of a significant result, the number of potentially missing studies needed to render the *p* value nonsignificant was calculated by the “fail-safe *N*” approach as another indicator of publication bias [37].

## 3. Results

**Figure 1:**
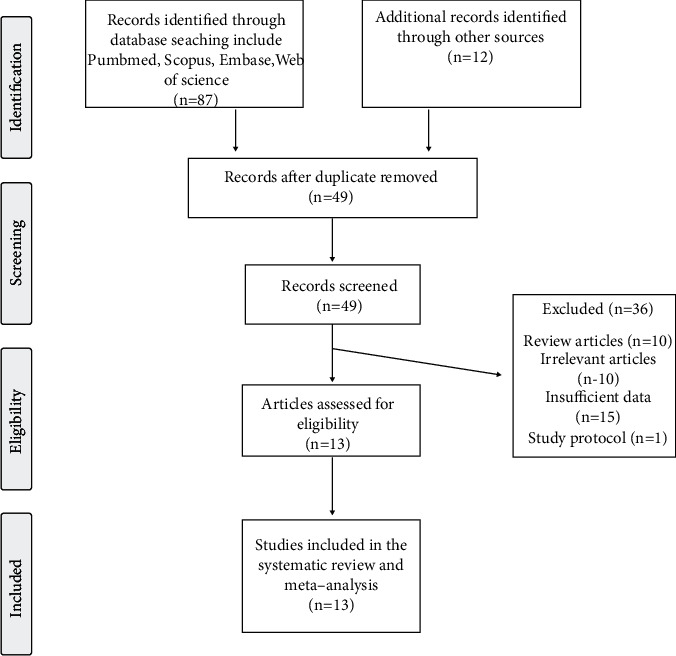
Flow chart of studies identified and included in the meta-analysis.

A thorough database search identified 99 published papers, 49 of which were directly related to the topic of this meta-analysis. After careful consideration, 36 studies were excluded: 10 studies were reviews, 10 studies did not meet the inclusion criteria, 15 studies did not report sufficient data, and one was a study protocol only. Therefore, 13 studies which evaluated the levels of Lp(a) before and after bariatric surgery were included (Table 1). The study selection process is shown in Figure 1. Assessment of risk of bias in the included studies is summarized in Table 2. Risk assessments for all studies were deemed to have high risk of bias.

### 3.1. Quality Assessment of the Included Studies

Because most of the studies did not have a control group, they were not evaluated for selection of controls, definition of controls, comparability, the same method of ascertainment, and nonresponse rate. However, all studies which were included met the ascertainment of exposure criteria. Table 2 shows the details of quality assessment.

### 3.2. Methods for Measuring Lp(a)

In most of the included studies, serum Lp(a) was assessed using the enzyme-linked immunosorbent assay (ELISA) [46]. One study used standard colorimetric methods using the Architect ci8200 analyzer (Abbot Diagnostics, Berkshire, UK [44]. One study used particle-enhanced immunoturbidimetry (Diagnostic System, GmbH, Holzheim, Germany) [43] while another used the turbidimetric assay using the Tina-quant Lipoprotein (a) Gen.2 system (Cobas Integra 400/800, Roche Diagnostics, Mannheim, Germany) [47]. Another study assessed Lp(a) by chemiluminescent immunoassays [50], and one study used the Cobas Mira Plus (Roche Diagnostics) analyzer [38]. In seven studies, the method was not specifically mentioned [39–42, 45, 48, 49].

### 3.3. Effects of Bariatric Surgery on Circulating Concentrations of Lp(a)

**Figure 2:**
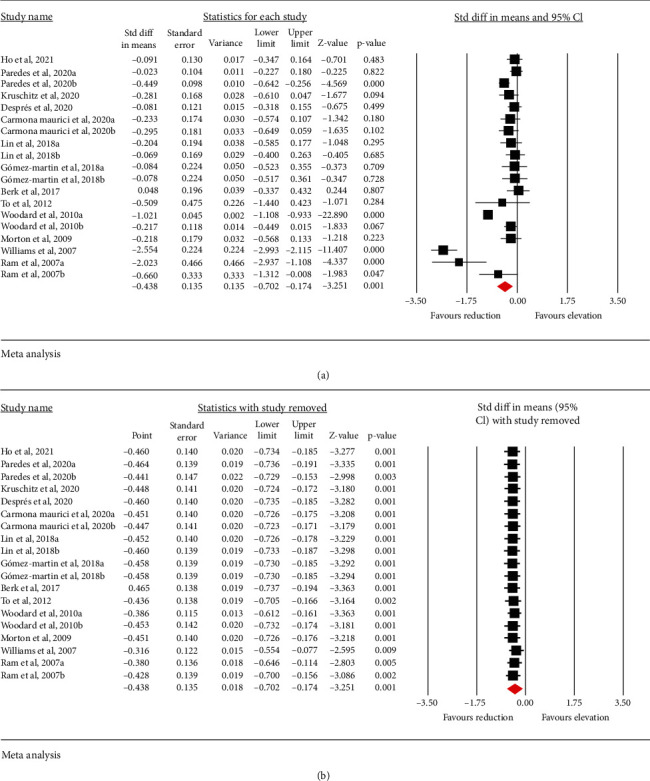
(a) Forest plot displaying weighted mean difference (SMD) and 95% confidence intervals (CI) for the effect of bariatric surgery on Lp(a). (b) Leave-one-out sensitivity analyses for the effect of bariatric surgery on Lp(a).

Meta-analysis of 13 studies including 1551 subjects showed a significant decrease of circulating Lp(a) after bariatric surgery (SMD: -0.438, 95% CI: -0.702, -0.174, *p* < 0.001, *I*^2^: 94.05%) (Figure 2(a)). The reduction in circulating Lp(a) was robust in the leave-one-out sensitivity analysis (Figure 2(b)).

### 3.4. Effects of Bariatric Surgery on BMI and Circulating Concentrations of LDL-C, HDL-C, and oxLDL

**Figure 3:**
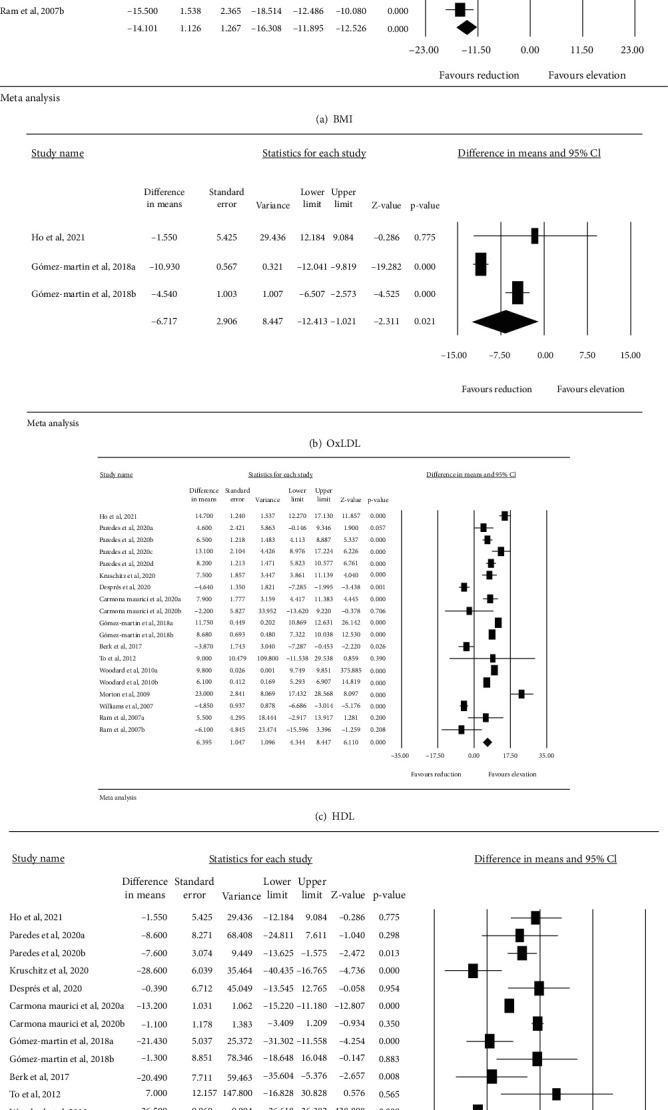
Effects of bariatric surgery on BMI and circulating concentrations of LDL-C, HDL-C, and oxLDL.

BMI in 12 studies including 1597 subjects significantly decreased after bariatric surgery (WMD: -14.101 kg/m^2^, 95% CI: -16.308, -11.895, *p* < 0.001, *I*^2^: 99.69%) (Figure 3(a)). Also, 3 studies including 99 subjects showed a significant decrease of circulating oxLDL after bariatric surgery (WMD: -6.717 mg/dL, 95% CI: -12.413, -1.021, *p* = 0.021, *I*^2^: 93.90%) (Figure 3(b)). Meta-analysis of 12 studies including 1530 subjects showed a significant increase of HDL-C after bariatric surgery (WMD: 7.390 mg/dL, 95% CI: 5.733, 9.046, *p* < 0.001, *I*^2^: 94.86%) as well as significant reduction in LDL-C levels (WMD: -14.166 mg/dL, 95% CI: -21.831, -6.502, *p* < 0.001, *I*^2^: 92.96%) (Figures 3(c) and 3(d)).

### 3.5. Metaregression

**Figure 4:**
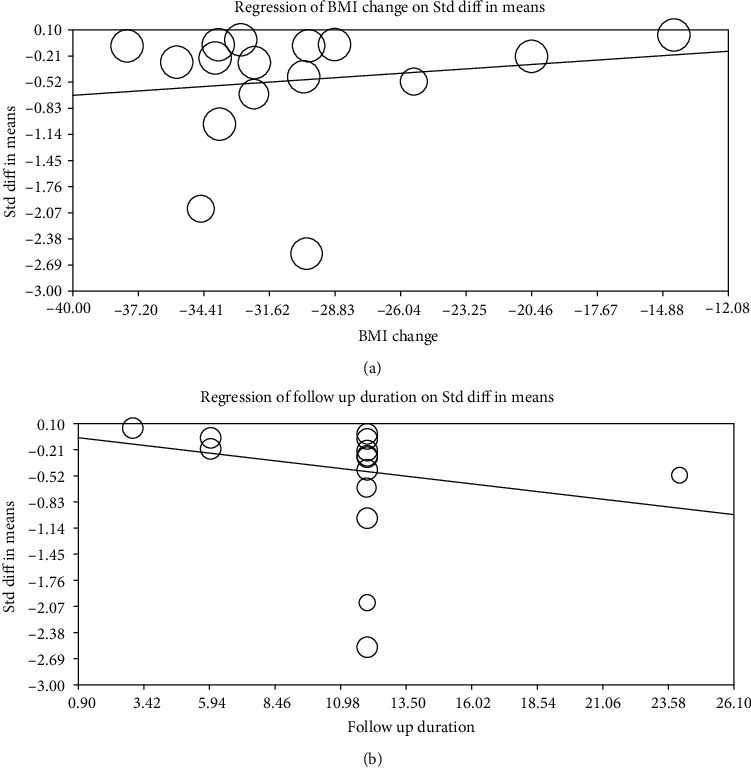
Random-effects metaregression for assessing the effect of % BMI change (a) and follow-up duration (b).

To investigate the impact of potential confounders on the Lp(a) lowering effect of bariatric surgery, random-effects metaregression was used. The results did not indicate any significant association between the changes in Lp(a) and percentage of BMI change (slope: 0.019; 95% CI: -0.037, 0.076; *p* = 0.507) or duration of follow-up (slope: -0.036; 95% CI: -0.112, 0.040; *p* = 0.355).  Figure 4 is shown.

### 3.6. Publication Bias

**Figure 5:**
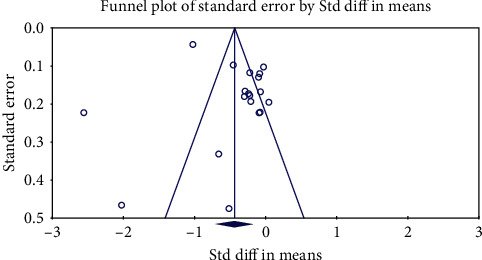
Funnel plot detailing publication bias in the studies reporting the effect of bariatric surgery on Lp(a).

Figure 5shows a funnel plot to evaluate publication bias across studies included in the meta-analysis. Egger's linear regression test (intercept = 2.935, standard error = 1.62; 95% CI = −0.498, 6.370, *t* = 1.803, df = 17, two-tailed *p* = 0.089) and Begg's rank correlation test (Kendall's Tau with continuity correction = −0.257, *z* = 1.57, two-tailed *p* = 0.115) did not indicate the presence of publication bias in this meta-analysis of bariatric surgery effects on circulating Lp(a). Trim-and-fill analysis indicated that among all papers included in this meta-analysis, there could be five missing studies. The “fail-safe *N*” test showed that 842 missing studies were required to reduce the effect size to a nonsignificant (*p* < 0.001) value. Statistical heterogeneity was observed as Cochran's *Q*-test and *I*^2^ (*p* < 0.05 and *I*^2^ > 50%, respectively).

## 4. Discussion

This meta-analysis showed a significant reduction in circulating Lp(a) levels after bariatric surgery. Several meta-analyses have analyzed these effects particularly in patients with type 2 diabetes. Favourable effects have been shown on serum triglycerides, total cholesterol, LDL-C, and HDL-C concentrations following bariatric surgery depending on its type and the anatomic alterations unique to each procedure [51–54].

Although an elevated Lp(a) level is independently associated with the incidence of cardiovascular events in the general population and it is an established predictor of cardiovascular events in patients with ASCVD [55, 56], the possibilities of decreasing elevated Lp(a) are still quite limited [57–63]. Unlike LDL-C and triglycerides, Lp(a) is relatively refractory to diet [64], lifestyle changes [65], and most drug interventions. Among medications, statins could increase plasma Lp(a) levels [66]. However, a decrease in Lp(a) concentrations can be achieved to a certain extent with niacin [67] which is not widely available. Mipomersen, an antisense oligonucleotide against apolipoprotein B [68–71], had the potential benefit to reduce Lp(a) by 20%, but this drug is approved by the FDA only as an orphan drug for homozygous familial hypercholesterolemia [72]. Currently, the only drugs on the market which can decrease Lp(a) significantly (about 25%) are proprotein subtilisin/kexin type 9 (PCSK9) inhibitors—alirocumab and evolocumab [73, 74]. A post hoc analysis of the Odyssey outcomes study suggested that a part of the benefits of alirocumab in reducing ASCVD events could be ascribed to its Lp(a) lowering effects, mainly on a subgroup of patients with high Lp(a) levels that had a recent myocardial infarction [75]. However, decreasing Lp(a) is not commonly accepted as an indication for use of PCSK9 inhibitors. Recently, data from a phase 2b trial with pelacarsen (an apo (a) antisense oligonucleotide) have attracted significant interest [76].

In this meta-analysis, bariatric surgery was shown to reduce Lp(a) levels despite the heterogeneity of the included studies. It has to be stressed that there was no association between changes in Lp(a) levels and weight loss or follow-up duration. An important question is: what mechanisms may cause these findings? One possibility is the consistent reduction in the obesity proinflammatory state indicated by lower levels of C reactive protein [77] and interleukin-6 (IL-6) after bariatric surgery [78] . Also, the *LPA* gene promoter contains IL-6-responsive elements consistent with Lp(a) acute phase response of apo (a) [79]. However, further studies are necessary to prove this hypothesis.

Previous findings on the reduction of LDL-C and atherogenic dyslipidemia [10–12] and Lp(a) as shown in this meta-analysis may explain the beneficial effects of bariatric surgery on individuals at high risk of ASCVD and mortality. However, this needs to be verified [3]. Moreover, it has been estimated that the magnitude of reduction required to achieve an ASCVD benefit is roughly 55 mg/dL [80]. Therefore, it has to be further explored whether Lp(a) reduction with bariatric surgery is of clinical relevance.

One of the limitations of this study was that the methods for measuring Lp(a) concentrations in some studies were different, and this might explain the heterogeneity in our findings. However, using SMD as the summary statistic in this meta-analysis could reduce this error. Indeed, because of the structural properties of Lp(a), none of the available commercial assays for Lp(a) quantification is 100% inherently isoform-sensitive [81]. We were also not able to establish the contribution of either apo (a) isoform size or variants in *LPA* gene [82]. Besides, some studies had no control group; some had small groups of patients or were not randomized. However, the results were still robust after the leave-one-out sensitivity analysis. Lp(a) has very little variability in different measures in individuals with stable health conditions [83]. We were also not able to evaluate the absolute reductions in Lp(a) and whether the effects were greater in patients with elevated Lp(a) levels. Finally, we were also unable to determine the specific impact of different bariatric surgery techniques, which may produce significantly stronger or weaker responses.

## 5. Conclusion

Obesity is associated with an increased ASCVD risk, and this association is consistent between sexes and across different parts of the world. This meta-analysis suggests that bariatric surgery significantly decreases circulating Lp(a) concentrations. Since elevated Lp(a) is independently associated with ASCVD, the results of this study may have clinical implications for severely obese individuals with high cardiovascular risk. However, it is worth mentioning that the estimates of the magnitude reduction required to achieve an ASCVD benefit are roughly 55 mg/dL [80].

## Figures and Tables

**Table 1:** Characteristics of studies measuring Lp(a).

Study year	Study design	Baseline Lp(a)	Follow-up	Treatment	Control	Clinical outcome	Patients	No. of patients
						Lp(a)		
Ram et al., 2007 [38]	Prospective study	30.30 ± 3.65	3 months 12 months	SRVG	**—**	Significant decrease in Lp(a) levels in both groups	Women with obesity Men with obesity	14 11

Williams et al., 2007 [39]	Prospective study	137.61 ± 45.90	3 months 6 months 12 months	RYGB	**—**	Significant decrease in Lp(a) levels	Patients with obesity	121 103 85

Morton and Boussard, 2009 [40]	Prospective study	14.00 ± 3.65	12 months	LRYGB	**—**	Significant decrease in Lp(a) levels	Adolescents with obesity	32

Woodard et al., 2010 [41]	Prospective study	32.20 ± 2.40 35.40 ± 8.20	12 months	RYGB LAGB	—	Unchanged	Patients with obesity	765 73

To et al., 2012 [42]	Retrospective study	35.00 ± 36.00	6 months 12 months 24 months	LSG	**—**	Significant decrease in Lp(a) levels at 12 months	Patients with obesity	52 39 5

Berk et al., 2017 [43]	Prospective study	88.88 ± 189.62	3 months	RYGB or gastric banding	Obese individuals without type 2 diabetes (dietary intervention)	Unchanged	Patients with obesity without T2DM	26

Gómez-Martin et al., 2017 [44]	Prospective study	40.00 ± 39.00 43.00 ± 64.00	6 months 12 months	LRYGB SG	Women matched for age and cardiovascular risk (diet and lifestyle modification)	Unchanged	Women with obesity	20 20

Lin et al., 2018 [45]	Prospective study	34.25 ± 59.03 34.25 ± 26.67	1 month 6 months	RYGB SG	**—**	Significant decrease in Lp(a) levels Significant decrease in Lp(a) levels at 1 month	Premenopausal women	27 35

Carmona-Maurici et al., 2020 [46]	Prospective study	258.17 ± 377.96 420.77 ± 462.56	6 months 12 months	LRYGB or SG	—	Significant decrease in Lp(a) levels at 12 months in both groups	Patients with obesity without plaque Patients with obesity with plaque	34 32

Després et al., 2020 [47]	Prospective study	69.50 ± 411.16	24 hours 5 days 6 months 12 months	Biliopancreatic diversion with duodenal switch (BPD-DS)	—	Significant increase in Lp(a) levels at 5 days Significant decrease in Lp(a) levels at 6 months	Patients with obesity	69

Kruschitz et al., 2020 [48]	Clinical trial	56.40 ± 91.60	1 month 6 months 12 months	Laparoscopic one anastomosis gastric bypass	**—**	Significant decrease in Lp(a) levels	Patients with obesity, serum 25(OH)D concentrations of <75 nmol/L	50 43 37

Paredes et al., 2020 [49]	Retrospective study	42.76 ± 126.82	12 months	SG	**—**	Significant decrease in Lp(a) levels Unchanged	Patients without metabolic syndrome Patients without metabolic syndrome	114 94

Ho et al., 2021 [50]	Prospective study	40.97 ± 155.40	6 months 12 months	RYGB or SG or omega loop bypass	Medical weight management	Significant increase in Lp(a) levels	Patients with obesity	59

LRYGB: laparoscopic Roux-en-Y gastric bypass; LAGB: laparoscopic adjustable gastric banding; LSG: laparoscopic sleeve gastrectomy; SRVG: silastic ring vertical gastroplasty; RYGB: Roux-en-Y gastric bypass; SG: sleeve gastrectomy.

**Table 2:** Quality of bias assessment of the included studies according to the Newcastle-Ottawa scale.

Study	Selection				Comparability^†^	Exposure		
	Case definition	Representativeness of the cases	Selection of controls	Definition of controls	Comparability of cases and controls	Ascertainment of exposure	Same method of ascertainment	Nonresponse rate
Berk et al., 2017	—	—	—	∗	∗	∗	∗	—
Després et al., 2020	—	—	—	—	—	∗	—	—
Ram et al., 2007	—	—	—	—	—	∗	—	—
Gómez-Martin et al., 2018	∗	—	—	—	∗	∗	∗	∗
Ho et al., 2021	—	—	—	—	—	∗	∗	—
Carmona-Maurici et al., 2020	∗	—	—	∗	∗	∗	∗	—
Kruschitz et al., 2020	—	—	—	—	∗	∗	∗	—
Lin et al., 2018	—	—	—	—	∗	∗	∗	—
Paredes et al., 2020	—	—	—	∗	∗	∗	∗	—
To et al., 2012	—	—	—	—	—	∗	—	—
Williams et al., 2007	—	—	—	—	—	∗	—	—
Woodard et al., 2010	—	∗	—	—	—	∗	—	—
Morton et al., 2009	∗	—	—	—	—	∗	∗	—

^†^Only for comparability, a maximum of two stars can be given.

## Data Availability

There is no primary data associated with this study.
